# Metagenomic analysis of gut microbiome illuminates the mechanisms and evolution of lignocellulose degradation in mangrove herbivorous crabs

**DOI:** 10.1186/s12866-024-03209-4

**Published:** 2024-02-13

**Authors:** Tom Kwok Lun Hui, Irene Ching Nam Lo, Karen Ka Wing Wong, Chandler Tsz To Tsang, Ling Ming Tsang

**Affiliations:** Simon F. S. Li Marine Science Laboratory, School of Life Sciences, The Chinese University of Hong Kong, Shatin, Hong Kong China

**Keywords:** Crustacea, Microbiome, Symbiosis, Lignocellulose degradation, CAZyme, Terrestrialization, Mangrove forest

## Abstract

**Background:**

Sesarmid crabs dominate mangrove habitats as the major primary consumers, which facilitates the trophic link and nutrient recycling in the ecosystem. Therefore, the adaptations and mechanisms of sesarmid crabs to herbivory are not only crucial to terrestrialization and its evolutionary success, but also to the healthy functioning of mangrove ecosystems. Although endogenous cellulase expressions were reported in crabs, it remains unknown if endogenous enzymes alone can complete the whole lignocellulolytic pathway, or if they also depend on the contribution from the intestinal microbiome. We attempt to investigate the role of gut symbiotic microbes of mangrove-feeding sesarmid crabs in plant digestion using a comparative metagenomic approach.

**Results:**

Metagenomics analyses on 43 crab gut samples from 23 species of mangrove crabs with different dietary preferences revealed a wide coverage of 127 CAZy families and nine KOs targeting lignocellulose and their derivatives in all species analyzed, including predominantly carnivorous species, suggesting the crab gut microbiomes have lignocellulolytic capacity regardless of dietary preference. Microbial cellulase, hemicellulase and pectinase genes in herbivorous and detritivorous crabs were differentially more abundant when compared to omnivorous and carnivorous crabs, indicating the importance of gut symbionts in lignocellulose degradation and the enrichment of lignocellulolytic microbes in response to diet with higher lignocellulose content. Herbivorous and detritivorous crabs showed highly similar CAZyme composition despite dissimilarities in taxonomic profiles observed in both groups, suggesting a stronger selection force on gut microbiota by functional capacity than by taxonomy. The gut microbiota in herbivorous sesarmid crabs were also enriched with nitrogen reduction and fixation genes, implying possible roles of gut microbiota in supplementing nitrogen that is deficient in plant diet.

**Conclusions:**

Endosymbiotic microbes play an important role in lignocellulose degradation in most crab species. Their abundance is strongly correlated with dietary preference, and they are highly enriched in herbivorous sesarmids, thus enhancing their capacity in digesting mangrove leaves. Dietary preference is a stronger driver in determining the microbial CAZyme composition and taxonomic profile in the crab microbiome, resulting in functional redundancy of endosymbiotic microbes. Our results showed that crabs implement a mixed mode of digestion utilizing both endogenous and microbial enzymes in lignocellulose degradation, as observed in most of the more advanced herbivorous invertebrates.

**Supplementary Information:**

The online version contains supplementary material available at 10.1186/s12866-024-03209-4.

## Background

Mangrove forests are ecologically and economically highly important ecosystems. They yield primary production (in terms of above-ground net primary productivity) comparable to the species-rich rain forests [1, 2]. The high carbon sequestration rates make the mangal ecosystem one of the most efficient natural carbon sinks globally to mitigate anthropogenic carbon emissions and climate change [3, 4]. It is, however, intriguing that few animals have evolved to exploit this abundant carbon source directly. One-third of the mangrove primary production is discarded in the form of leaf litter [5], which is mostly utilized by mangrove herbivorous invertebrates dominating the habitat, the sesarmid crabs (Brachyura: Thoracotremata: Sesarmidae) [6, 7]. Mangrove crabs from other families are mainly detritivores that feed on organic matter (e.g., microphytobenthos and decay detritus) in sediments and process significantly less plant matter compared to sesarmids [6]. The diversity of sesarmid crabs is disproportionately high, comprising more than half of the 400 known mangrove crab species globally [8]. The herbivorous habit of sesarmids suggests that their capacity of lignocellulose degradation and utilization of mangrove leaves as a food source may be an important evolutionary innovation that has allowed them to better adapt to the mangrove environment and as a result, promote speciation.

The sesarmid crabs are in fact crucial to the functioning of a healthy mangrove forest ecosystem. By feeding on mangrove leaves and fecal deposition, they actively enhance nutrient recycling in the ecosystem, providing an important trophic link between producers and higher consumers [6, 9]. Their bioturbation activities via burrowing also substantially impact many important biogeochemical processes, and hence vegetation patterns [9, 10]. Sesarmid crabs can thus be regarded as key ecological engineers in mangrove forests. Therefore, how sesarmid crabs digest plant tissue is a major question; not only fundamental to the understanding of the drivers for adaptive radiation, but also providing important insights into the ecosystem functioning and future restoration of mangrove forests. However, it remains poorly understood how sesarmid crabs achieve lignocellulose degradation and obtain sufficient nutrients from a plant-dominant diet. Generally, on top of the physical digestion of plant cell wall fibers, complete lignocellulose degradation requires collaborative action of various classes of enzymes, including cellulases, hemicellulases, laccases and pectinases, each with their own subset of enzymes responsible for side-branch, backbone and oligosaccharide degradation of the respective lignocellulose component (see Supplementary Table S1 for a summary of enzymes involved in lignocellulose degradation and reviewed by [11, 12]). Majority of the herbivorous animals rely at least partially on the enzyme produced by endosymbiotic microbes in digesting plant fiber [13, 14]. Crustaceans can encode endogenous Carbohydrates Active enZymes (CAZymes) for lignocellulose degradation, which are hypothesized to be responsible for the degradation of algal cell wall, a major component in their omnivorous diet [15, 16]. Transcriptomic and enzyme assay studies have confirmed the production of endogenous cellulolytic enzymes in herbivorous decapod species [17–19], including sesarmid crabs [20, 21]. However, it remains largely unclear that if mangrove herbivorous crabs possess complete metabolic pathways to degrade lignocellulose, or if they require a symbiotic microbiome to complement the digestion process as the situation observed in terrestrial isopods [22] and termites [23–26], due to limited clues. The digestive tracts of crab species are short, even in herbivorous species, such that the passage time of food in their digestive system is believed to be insufficient for microbial fermentation and hence microorganisms present in decapod intestine are hypothesized to be not essential for digestion and nutrition [27]. However, the functional role and importance of symbiotic microbes in nutrition have seldom been systematically investigated in any crab. Metagenomes for the gut symbiont were only sequenced for a few species with commercial importance (e.g., Chinese mitten crab *Eriocheir sinensis* [28, 29]; mud crab *Scylla* spp [30, 31]). The gut bacterial communities were characterized on three mangrove sesarmid crabs using 16S amplicon sequencing and microbes with capacity of cellulose degradation and nitrogen fixation were found [32]. This is consistent with the previous detection of sediment bacteria inside the fiddler crab intestines with possible nitrogen fixing capabilities [33], suggesting that the gut microbes may contribute to both digestion and nutrition of the host crabs.

In the present study, we attempt to characterize the gut bacterial metabolic potentials on lignocellulose degradation using metagenomic sequencing to validate the hypothesis that endosymbiotic cellulolytic microbes play an important role in lignocellulose degradation in mangrove herbivorous sesarmid crab species. We further compare the microbiome of other mangrove crab species with different dietary preferences from other families to determine whether herbivorous crabs are enriched in lignocellulose degrading CAZy families, KOs, or related bacterial taxa. We hypothesize that the dietary need of crabs is strongly correlated to the abundance of lignocellulolytic bacteria, and they are highly enriched in herbivorous sesarmids compared to carnivores and omnivores, thus enhancing their capacity for digestion of mangrove leaves. Furthermore, the contribution of gut symbiotic microbes to the nitrogen economy of the crabs will also be explored.

## Methods

### Sample collection

Specimens of 23 crab species in nine brachyuran families were collected from mangroves and intertidal habitats in Hong Kong from 2019 to 2021 (Table 1; Supplementary Fig. 1). Species were categorized as herbivores (H), detritivores (D), omnivores (O) and carnivores (C) according to their dietary habits reported in previous studies. Crabs that actively cut and consume leaf litter were classified as herbivores (e.g., Sesarmidae crabs). Crabs that sieve organic matters, microphytobenthos and detritus from sediments (e.g., fiddler crabs and *Metaplax*) were grouped as detritivores while crabs mainly hunt or scavenge for animal remains and occasionally scrape algae as food source were defined as omnivores (e.g., *Hemigrapsus penicillatus*, *Ocypode ceratophthalmus*, *Metopograpsus frontalis*, and Xanthidae crabs). Portunidae and Oziidae crabs that actively hunt for animal tissues and less commonly ingest algal or plant matters were classified as carnivores. Details of the diet content and taxonomic information of the target species are listed in Table 1. Only male crabs were collected to avoid variance from sex and crabs were anesthetized on ice immediately upon collection to minimize changes in microbial composition during transportation. Upon arrival at the laboratory, specimens were dissected and the contents in midgut and hindgut with the enveloping peritrophic membrane that harbors a high density of gut microbes were isolated and preserved in 95% ethanol. Gut tissues were discarded to minimize contamination from host cells. For each species, gut contents of equal weight from three individuals collected at the same location and date were pooled and homogenized as one biological sample to cover individual variations.

### DNA extraction and metagenome sequencing

DNA was extracted from 0.6 g of each pooled biological sample using a protocol combining the use of QIAamp PowerFecal DNA Kit (QIAGEN) and DNA fragment size selection with SPRI magnetic beads (Beckman Coulter). Initial cell lysis and inhibitor removal steps were performed using the QIAamp PowerFecal DNA Kit following the manufacturer’s instructions. The column binding step was replaced by 0.65x SPRI beads for DNA fragment size selection. The resultant solutions were mixed carefully and then split into microcentrifuge tubes and stood on a magnetic rack for separation of the beads with DNA and the buffer. 500 µl of Solution C5 (washing buffer) of the QIAamp PowerFecal DNA kit was added to wash the beads twice, and the Solution C5 was then aspirated. The beads with DNA samples were allowed to air-dry for five minutes for the removal of excess ethanol. 54 µl of ddH2O was then added to the beads and stood for five minutes before elution. The eluates were collected on a magnetic rack and transferred to new microcentrifuge tubes. Samples with high DNA purity and majority of the fragments > 500 bps were sent to a commercial biotechnology company (Novogene, China) for quantity and purity measurements using Agilent 2100. Samples passing QC were used for metagenomic library preparation and sequenced on the Illumina NovaSeq 6000 Platform targeting around 30Gb output data of 150 bp paired-end raw reads (approximately 200 M reads) per sample.

**Table 1:** Mangrove crab species analyzed in the current study and their major diet content reported in previous studies. A lower trophic position reflects a higher dietary proportion of primary producers and a tendency of herbivory, while a higher trophic position reflects a tendency of predation or microbivory [81]

Dietary group	Family	Species	Diet contents	Trophic position [81]	References	
Herbivore	Sesarmidae	*Clistocoeloma villosum*	Mangrove litter, suspended organic matter^#^		[88]	
		*Episesarma versicolor*	Detritus, bark or root, leaf, algae	1.9 ± 0.2	[80, 89]	
		*Fasciarma fasiatum*	Plant, organic matter, animal matter^#^		-	
		*Orisarma dehaani*	Plant, organic matter, animal matter		[90]	
		*Orisarma intermedium*	Leaf litter, diatoms		[91]	
		*Orisarma sinensis*	Leaf litter, diatoms^#^		[91]	
		*Parasesarma affine*	Leaf litter, diatoms	2.1 ± 0.3	[91]	
		*Parasesarma continentale*	Algae, leaf litter	1.8 ± 0.2	[92]	
Detritivore	Macrophthalmidae	*Macrophthalmus definitus*	Sediment organic material	1.6 ± 0.2^*^	[93]	
	Mictyridae	*Mictyris brevidactylus*	Sediment organic material		[94]	
	Ocypodidae	*Gelasimus borealis*	Microphytobenthos, microheterotrophs, bacteria, detrital matter	1.9 ± 0.2	[95]	
		*Paraleptuca splendida*	Microphytobenthos, microheterotrophs, bacteria, detrital matter	2.3 ± 0.3	[95]	
		*Tubuca arcuata*	Microphytobenthos, microheterotrophs, bacteria, detrital matter		[95]	
	Varunidae	*Metaplax longipes*	Detritus, bark or root, sand, algae^#^	2.4 ± 0.3	[89]	
		*Metaplax tredecim*	Detritus, bark or root, sand, algae	2.4 ± 0.3^*^	[89]	
Omnivore	Grapsidae	*Metopograpsus frontalis*	Algae, leaf litter, crabs, gastropods, fish	2.3 ± 0.3^*^	[92]	
	Ocypodidae	*Ocypode ceratophthalmus*	Sediment organic material, animal carrion		[96]	
	Varunidae	*Hemigrapsus penicillatus*	Bivalves, algae, gastropods, crustaceans, polychaetes^#^		[97]	
	Xanthidae	*Etisus laevimanus*	Algae		[98]	
		*Leptodius affinis*	Algae		[98]	
Carnivore	Oziidae	*Epixanthus frontalis*	Crabs, gastropods^#^		[72]	
	Portunidae	*Scylla paramamosain*	Fish, mollusks, fish, shrimp		[70]	
		*Thranita danae*	Bivalves, gastropods, crabs, algae^#^		[71]	

#   Data from crabs from same genus/family

### Functional & taxonomic annotations and diversity indices calculation

For the downstream data analyses, default parameters were used unless otherwise specified. Adapter sequences, duplications, and reads of quality below Q15 were trimmed using fastp v.0.22.0 [34] with *--dedup* flag enabled for deduplication. Trimmed reads of each species were further processed and annotated using an integrated pipeline SqueezeMeta v1.4.0 [35] with *-extdb* flag to add annotation from CAZy database. Clean reads were co-assembled into contigs from samples of the same species by MEGAHIT v.1.2.9 [36] and the Open Reading Frames (ORFs) were predicted using Prodigal v2.6.3 [37]. For each sample, clean reads were also mapped to contigs using Bowtie2 v2.3.4.1 [38]. Raw counts for each ORF, gene, and contig in each sample were obtained for downstream analyses. The ORFs and contigs were mapped and annotated to the latest publicly available KEGG Orthology (KO) (release v58) [39] and CAZy databases (release V10, 07292021) [40]. KO entries were represented in terms of functional orthologs and integrated into molecular networks and pathways, while CAZy database has a more comprehensive coverage on lignocellulose degrading enzymes and gene features. Combining annotations from both databases would enhance the recovery and identification of lignocellulolytic genes of interest. The taxonomic origin of the ORFs was searched against the RefSeq non-redundant protein (nr) databases (release 243) [41] using blastx mode of DIAMOND v2.0.8.146 [42]. ORFs of non-bacterial origin and samples with less than 1 M mapped prokaryotic reads were excluded from the subsequent analyses.

Chao1, Shannon and Simpson indices of each sample were calculated from raw bacterial counts at taxonomic levels from phylum to species using R package *phyloseq* [43]. Chao1 index reflected the corrected richness of observed taxa [44], while Shannon [45] and Simpson [46] indices revealed the evenness of taxa where dominant species were weighed more in Simpson index. Alpha diversity indices of different dietary groups were compared using Wilcoxon rank sum test with false discovery rate (FDR) correction to adjust for multiple comparisons. As Chao1 index was expressed as integers and the test cannot compute an exact *p*-value in case of ties, a conserved estimate was output instead before conducting FDR correction.

### Normalization and differential abundance analysis

Normalization of raw counts is essential to achieve meaningful cross-sample comparisons by eliminating the effects of individual variations in sequencing depth, composition, and gene length. Different normalization methods have been developed with the rapid increase in differential testing tools and studies using high throughput sequencing data. Despite high throughput sequencing data is generally considered compositional and similar statistic models were employed in the various differential testing tools [47], benchmarking studies showed different sensitivity and specificity across differential abundance analysis (DAA) tools in 16S datasets [48], shotgun metagenome datasets [49], and transcriptome datasets [50]. Thus, a consensus approach was adopted in the current study to make a conservative call for differentially abundant features with reproducible results across different DAA tools [48, 49]. Among the available tools, ALDEx2 [51] and ANCOM-BC [52] were used as they were reported to show lower FDR in the benchmark studies [53, 54]. DEseq2 [55] was also included for its popularity and the alternative normalization approach accounting for library size adopted by the tool. Median of ratio was used in DESeq2 for normalization while log-ratio transformations were used in ALDEx2 and ANCOM-BC, despite both approaches relying on the ratio of counts relative to the geometric mean abundance.

DAA was performed as pairwise comparisons among the four dietary groups (H, D, O and C), resulting in 12 pairs of comparisons for each set of features. Functional features were represented as CAZy families and KOs, while taxonomic features were compared at the phylum level because of its lower proportion of unclassified taxa as compared to lower taxonomic levels (see Results). For DAA of functional features, a set of 76 Universal Single Copy Genes for bacteria (USiCGs) were used as a reference for both library size and gene copy number [56]. While for taxonomic features, all phyla were used for normalization. Features with normalized counts < 1000 and existed in < 20% of samples were prefiltered out using *CoDaSeq* package [47] to reduce variance and false positives. Only features commonly identified by at least two tools were considered as differentially abundant (DA) in this study.

CAZy families and KO entries related to lignocellulose degradation reported in previous studies [11, 22, 57] listed in Supplementary Table S1 were analyzed. Another list of KO entries of nitrogen metabolism-related functions [58] listed in Supplementary Table S2 was also studied to investigate the capability of bacteria to recycle ammonia wastes and fix atmospheric nitrogen into utilizable amino acids. Log-adjusted abundances generated from ANCOM-BC were used as a representative for visualization of results in subsequent analyses with the associated statistics for all analytic tools shown in the corresponding comparison. Heatmaps displaying abundances of DA features were generated using *pheatmap* package [59], with the samples clustered according to the phylogenetic tree from Tsang, Schubart [60].

### Hierarchical clustering and principal coordinate analysis

Aitchison distances [61] among samples were calculated from normalized counts for each set of features. Samples were clustered according to the Aitchison distances using Ward’s method [62] and visualized as hierarchical clustering trees and Principal Coordinate Analysis (PCoA) plots to illustrate the (dis)similarity among samples. Statistical verification of the dissimilarity was conducted with pairwise permutational multivariate analysis of variance (PERMANOVA) comparisons of Aitchison distances across dietary groups using *pairwiseAdonis* package [63].

### Taxonomic origin of genes related to lignocellulose degradation and nitrogen metabolism

R package *SQMtools* [64] was used to identify the taxonomic origin of genes related to lignocellulose degradation and nitrogen metabolism. Taxonomic information was extracted from the annotated dataset of each species using the *subsetFun* and *subsetTax* functions of *SQMtools*. Counts transformed into relative abundances were allocated to the corresponding substrates and bacterial phyla. Adjacency matrices were then generated and visualized using Circos webtool [65].

## Results

A total of 43 samples from the 23 species have passed the quality filters with more than 1 M mapped prokaryotic reads. The number of herbivorous, detritivorous, omnivorous, and carnivorous crab samples successfully sequenced were 12, 18, 7 and 6, respectively (Supplementary Table S3). The sequencing metrics for individual metagenomes were summarized in Supplementary Table S3. The metagenomes had an average of 14,360,783 prokaryotic reads (ranging between 1,646,180 and 66,420,675). Percentages of prokaryotic reads varied from sample to sample, with the highest of 61.8% in one *Episesarma versicolor* sample to the lowest of 0.9% in *Macrophthalmus definitus*. Twenty-nine samples out of 43 in this study had lower than 10% of prokaryotic read identified. Herbivorous and detritivorous crabs generally had a lower average number of mapped prokaryotic reads (14.6 M and 6.8 M respectively), compared to 26.9 M in carnivorous and 22.6 M in omnivorous crabs. The reads were assembled into 33,557 to 9,668,764 contigs with an average of 5,017,671 per sample. The average N50 of the contigs was 784 (ranging from 499 to 1,483) and the N50 were also higher in carnivores (average = 1,177, range = 830 to 1,483) and omnivores (average = 882, range = 730 to 1,271) then that in herbivores (average = 763; range = 610 to 1,218) and detritivores (average = 629; range = 499 to 870). Raw read data of all metagenomes are available at NCBI Sequence Read Archive (SRA) under BioProject accession number PRJNA1017629 while metagenome assemblies and unnormalized read count tables for taxonomic and functional features are available at DRYAD under https://datadryad.org/stash/share/jGokZ8PmSAfDhjV_iAN4UgVWlgFqh4pqDGrEcY8GW30.

### Composition and diversity of mangrove crabs gut symbiotic bacteria

**Fig. 1:**
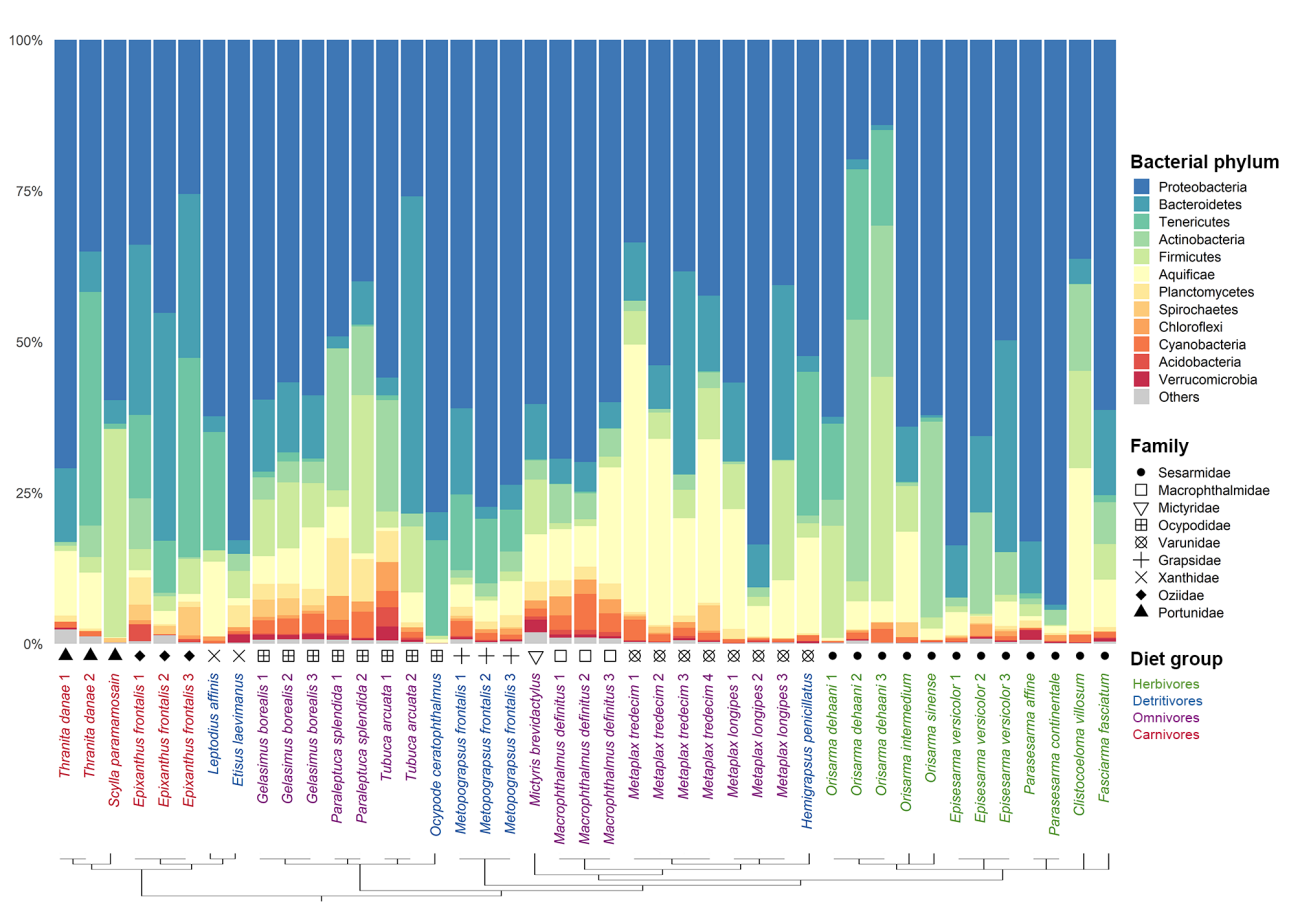
Relative abundances of bacterial phyla in each crab metagenome sample. Samples are clustered by phylogeny inferred in [59]. Sample names are colored according to the dietary groups and symbols correspond to the family. Bacterial phyla with relative abundances less than 1% in all samples are grouped as “Others”

**Fig. 2:**
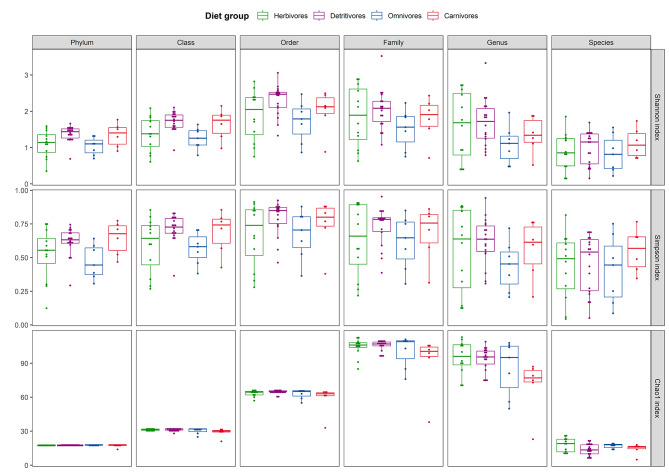
Distribution of alpha diversity metrics in terms of Shannon index, Simpson index and Chao1 index among different dietary groups on **(A)** phylum, **(B)** class, **(C)** order, **(D)** family, **(E)** genus and **(F)** species levels. The bold line within the box corresponds to the median of the group. The bottom and top of the box correspond to the 25th and 75th percentiles. The whiskers are drawn to the 10th and 90th percentiles. Each point represents a sample

Eighteen bacterial phyla were identified and contributed 81.2% of the annotated prokaryotic reads. The proportion of classified reads gradually decreased down taxonomic ranks, with only 6.2% of reads annotated to 28 taxa at the species level. Proteobacteria was the most dominant bacterial phylum in the samples with an average relative abundance of 56.1%, followed by Bacteroidetes (11.1%), Aquificae (8.49%), Firmicutes (6.80%), Actinobacteria (6.42%) and Tenericutes (5.72%) (Fig. 1). These six most abundant bacterial phyla constituted over 90% of the annotated reads in the crab metagenomes. Alpha-diversity metrics of the samples were compared across dietary groups from phylum to species levels to explore the richness and evenness of bacterial taxa identified (Fig. 2). At the phylum level, dietary groups D and O were significantly different in Shannon and Simpson indices, while a significant difference was only detected in the Shannon index at the class level (Supplementary Table S4). Despite a significant difference was observed between dietary groups D and C in Chao1 index at the genus level, the results need to be interpreted with caution given the high proportion of unclassified reads. Furthermore, no significant difference in alpha diversity indices was detected across crab families, which could be attributed to the relatively small sample sizes for most crab families analyzed to provide statistically meaningful results.

### Comparison of bacterial taxonomic profiles across dietary groups

**Fig. 3:**
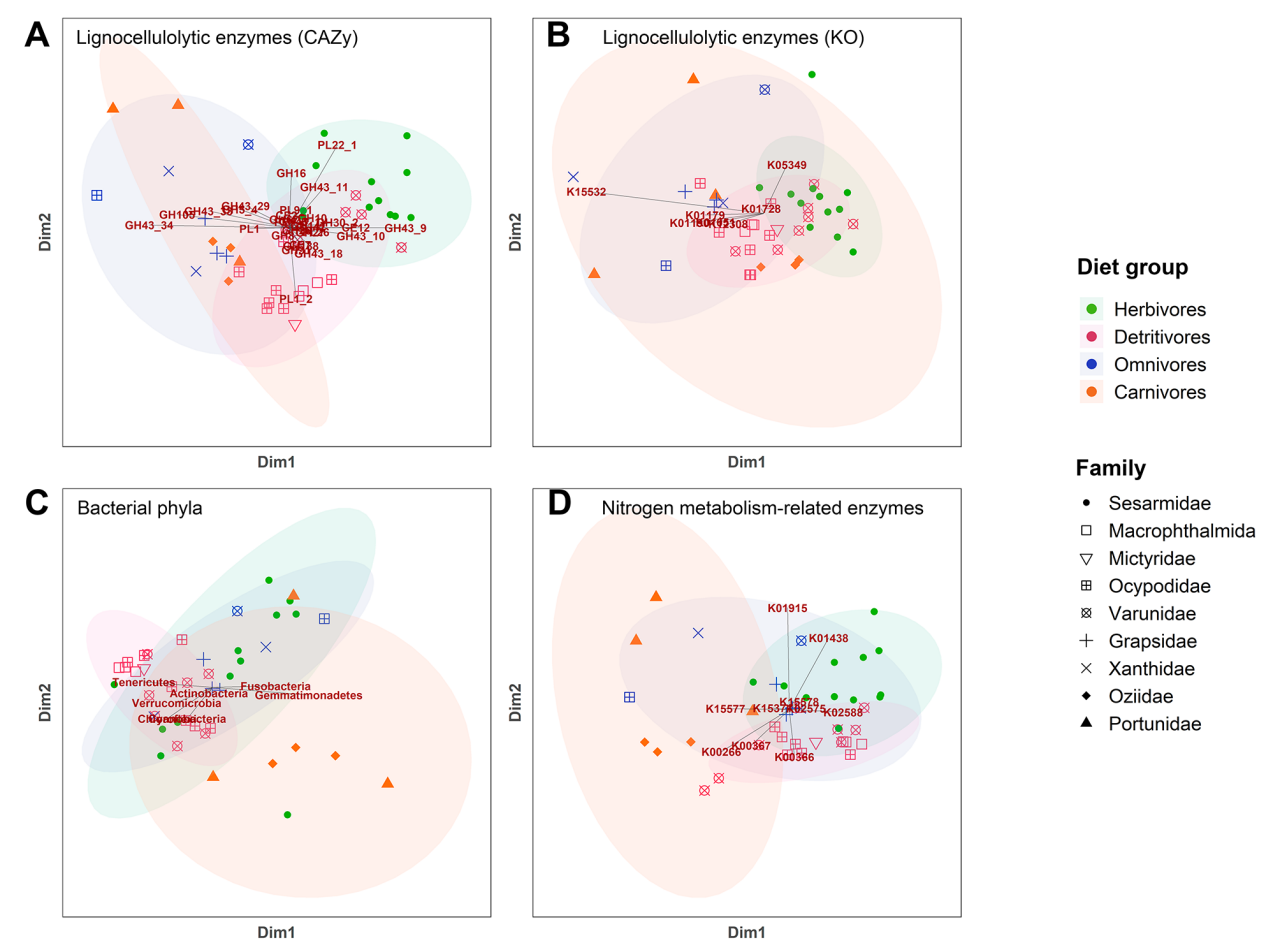
PCoA plots of the (dis)similarities among crab metagenome samples according to the matrix of different features. **(A)** Lignocellulolytic enzymes annotated to CAZy families, **(B)** lignocellulolytic enzymes annotated to KEGG Orthology (KO) entries, **(C)** bacterial phyla and **(D)** nitrogen metabolism-related enzymes annotated to KEGG Orthology entries. Each point corresponds to a sample and the symbol represents the family to which the sample belongs. Labels in red are features with significant differences in at least one pairwise comparison among the dietary groups identified with consensus from ALDEx2, ANCOM-BC and DESeq2

**Fig. 4:**
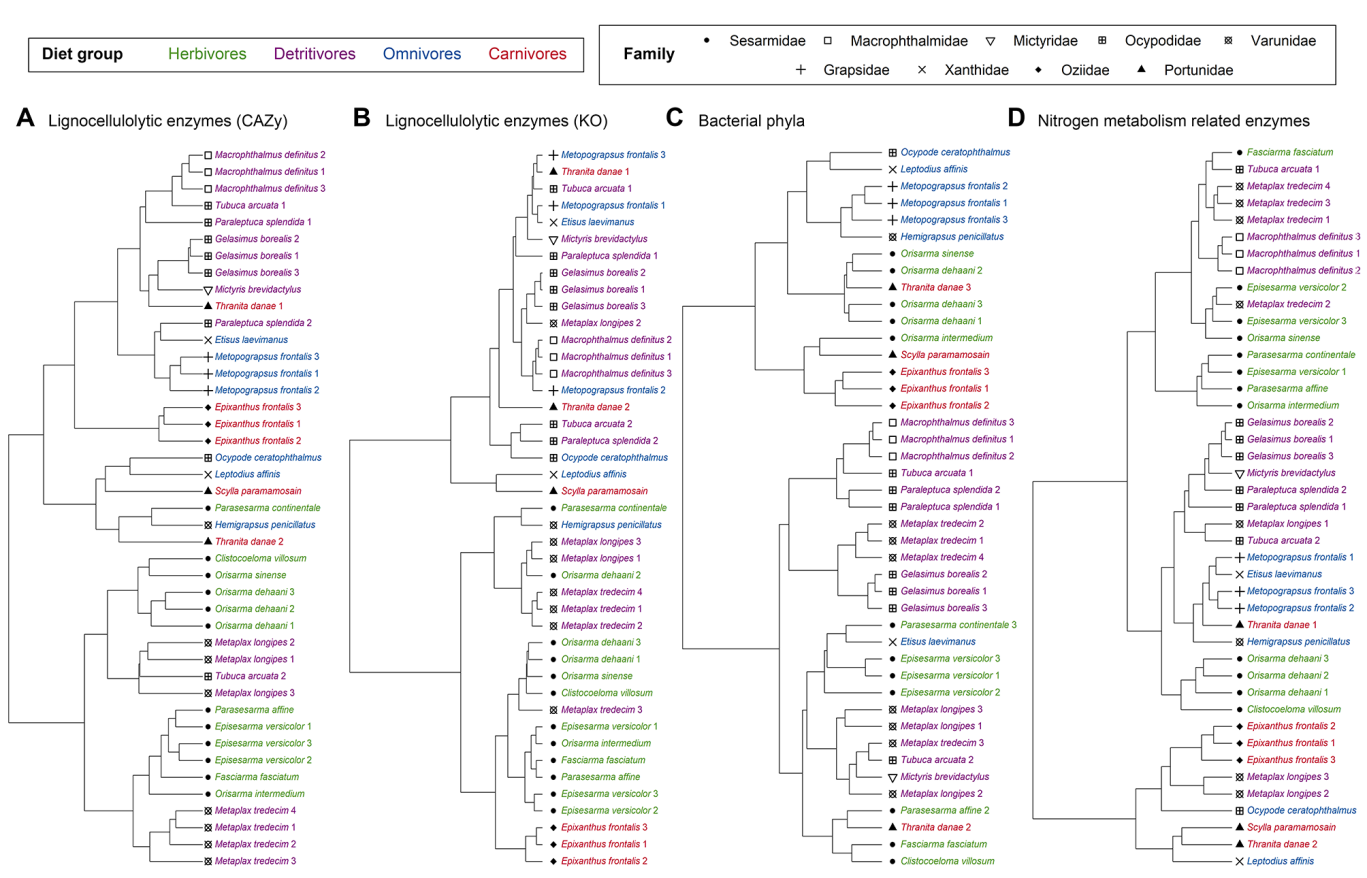
Hierarchical clustering trees of samples based on the abundance of different features. **(A)** Lignocellulolytic enzymes annotated to CAZy families, **(B)** lignocellulolytic enzymes annotated to KEGG Orthology (KO) entries, **(C)** bacterial phyla and **(D)** nitrogen metabolism-related enzymes annotated to KEGG Orthology entries. Sample names are colored according to the dietary groups and symbols correspond to the family

PCoA plot and hierarchical clustering tree based on bacterial taxonomic profiles of annotated phyla in the crab metagenomes showed largely congruent results and revealed that most samples from the same species shared similar prokaryotic profiles, though some individual variations were observed (Figs. 3 and 4). No distinct clustering pattern was observed based on the crab families alone in Sesarmidae, Varunidae and Ocypodidae where there were more than three species analyzed per family in the present attempt (Figs. 3C and 4C). The bacterial taxonomic profiles were more heterogenous within carnivorous and herbivorous crabs while omnivores and detritivores shared more similar bacterial communities with species having the same dietary preference, despite the hosts belongs to different crab families. All but one omnivorous species (*Etisus laevimanus*) grouped together in the hierarchical clustering tree, while detritivores formed a large assemblage together with a few herbivorous species. Concerning the similarity in profiles amongst dietary groups, the carnivorous crabs partially overlapped with groups H and O, which were largely intermingled in the PCoA plot. Detritivorous crabs also partially overlapped with H and O, but mostly separated from C. PERMANOVA analyses on the relative abundance of bacterial phyla showed that the prokaryotic compositions were significantly different in all comparisons among the four different dietary groups (FDR adjusted p-value < 0.05; Supplementary Table S5), despite the observed overlapping in the PCoA plot or cluster tree.

For the differentially abundant (DA) bacterial phyla detected by at least two of the DA testing tools, Fusobacteria was significantly more abundant in C than all other three dietary groups, while Tenericutes was more abundant in C than D and in O than D. Four phyla (Chloroflexi, Cyanobacteria, Gemmatimonadetes and Verrucomicrobia) were of higher abundances in D than C, in which Gemmatimonadetes was also more abundant in D than H. PCoA plot based only on the DA bacterial phyla identified recovered largely similar clustering pattern with that generated based on all bacteria taxa (Supplementary Fig. 2).

### Identification of microbial lignocellulose degrading CAZymes and enrichment across dietary groups

**Fig. 5:**
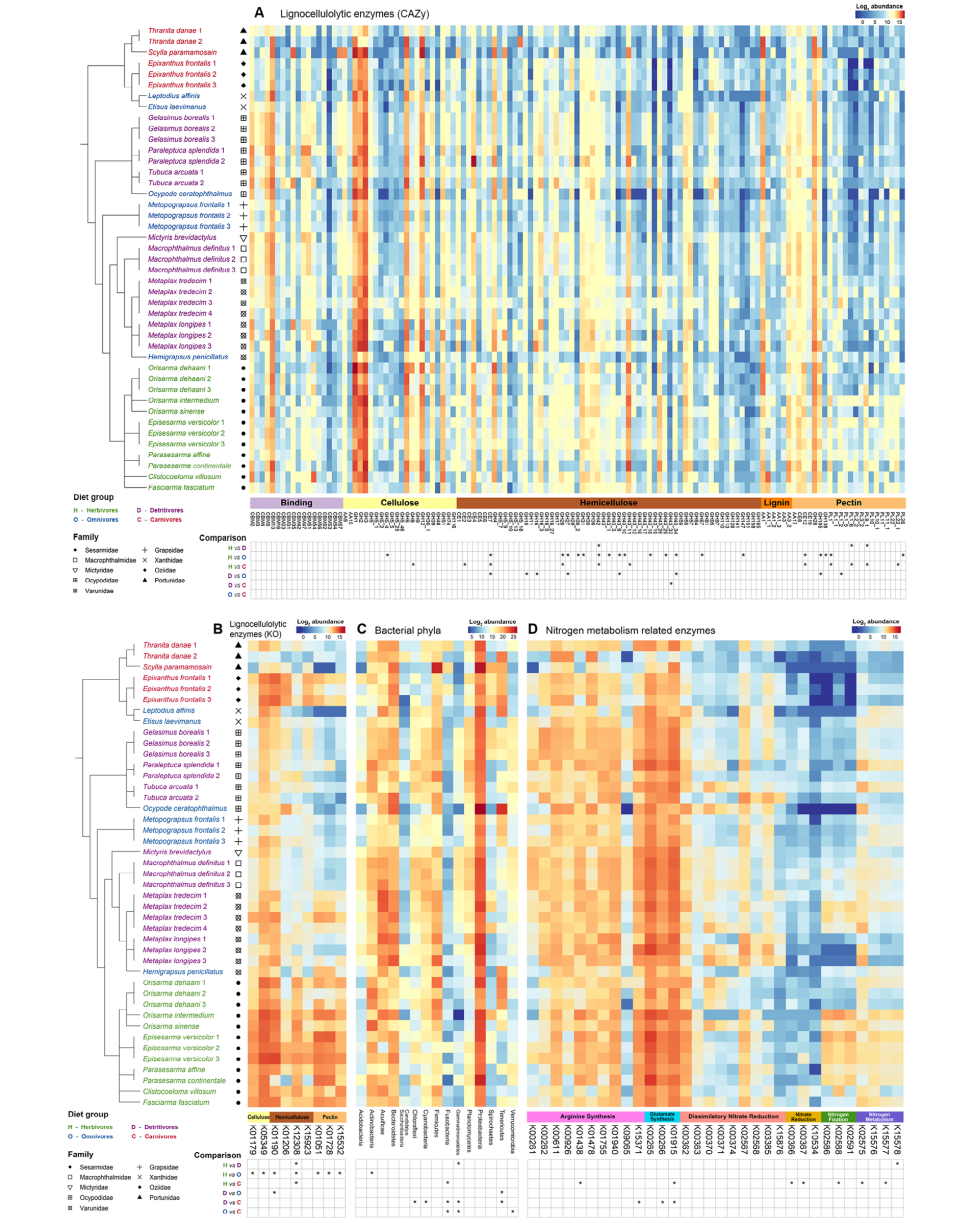
Heatmaps of log_2_ normalized abundances of different features. **(A)** Lignocellulolytic enzymes annotated to CAZy families, **(B)** lignocellulolytic enzymes annotated to KEGG Orthology entries, **(C)** bacterial phyla and **(D)** nitrogen metabolism-related enzymes annotated to KEGG Orthology (KO) entries. Samples are clustered according to phylogenetic relationship reported in Tsang et al. [60]. Sample names are colored according to the dietary groups and symbols correspond to the family. Features are arranged according to enzyme categories in A, B and D; and by alphabetical order in C. Differentially abundant features detected in pairwise comparisons with the consensus in at least two of the differential abundance testing tools (ALDEx2, ANCOM-BC and DESeq2) among dietary groups are denoted by asterisks in the table below the heatmap

**Fig. 6:**
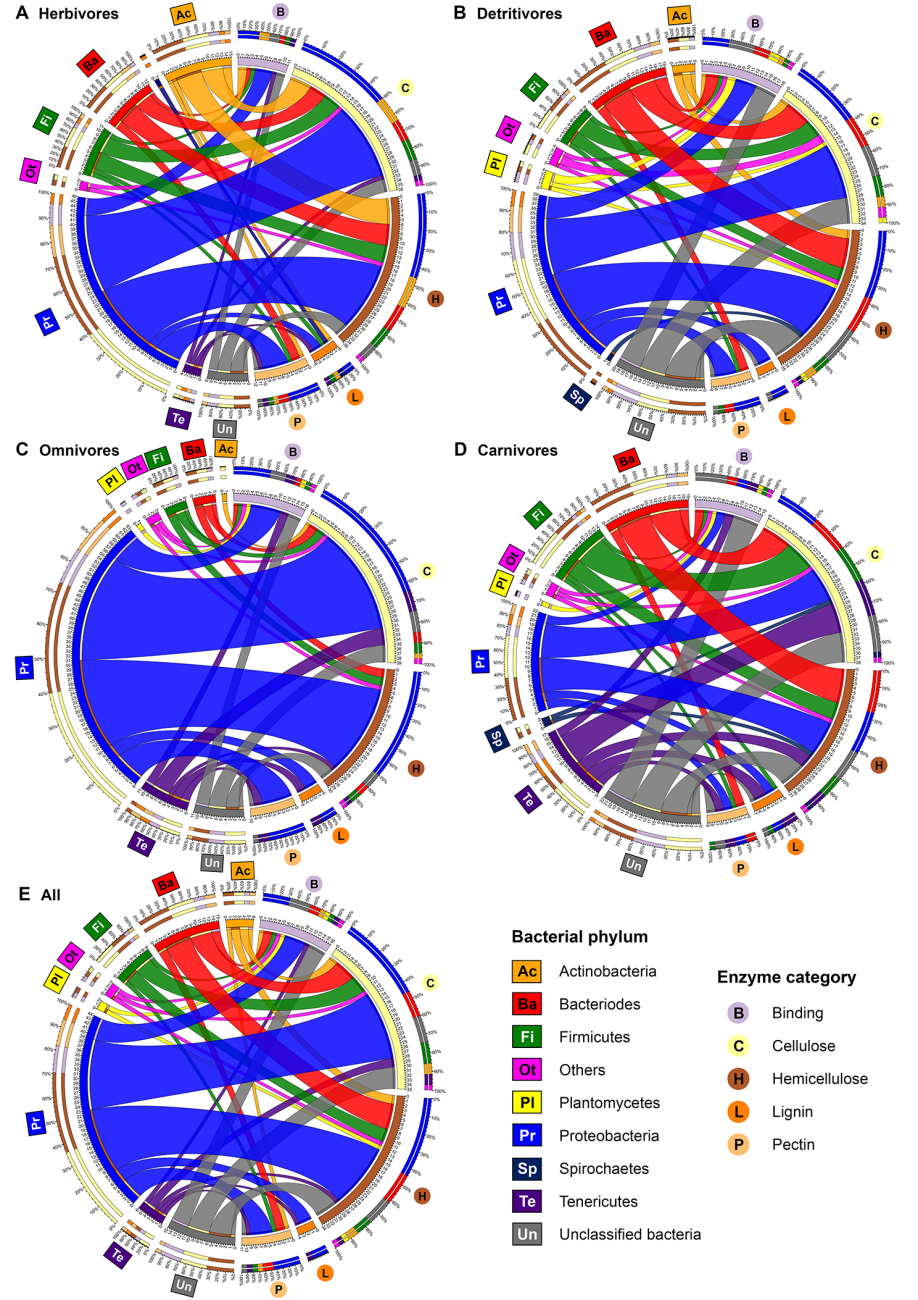
Chord diagrams showing the inter-relationship between bacterial phyla and enzyme categories in lignocellulolytic enzymes identified from either CAZy or KEGG Orthology databases in **(A)** herbivores (*n* = 12), **(B)** detritivores (*n* = 18), **(C)** omnivores (*n* = 7), **(D)** carnivores (*n* = 6) and **(E)** all samples (*n* = 43)

The prokaryotic contigs recovered a total of 127 out of 231 lignocellulose degradation related CAZy families, including 75 glycoside hydrolases (GH), ten carbohydrate esterases (CE), 15 polysaccharide lyases (PL), nine auxiliary activities (AA), and 18 of carbohydrate-binding modules (CBM) (Fig. 5A). For KO entries, nine out of 71 lignocellulose degradation related KOs were recovered (Fig. 5B). Enzymes responsible for the degradation of cellulose, hemicellulose, pectin and lignin composed of 35%, 34%, 11% and 4% of the reads, respectively, while 16% of reads were annotated to enzymes with binding function (Fig. 6). The 127 identified CAZymes were found in metagenomes of crabs from all four dietary groups. However, herbivorous crabs had the highest abundances of lignocellulose degrading genes followed by detritivorous crabs, despite the gene diversity being comparable among the four dietary groups. GH 1, 2, 3, 4, 6, 12, 28, 43_11, AA 1 and CBM 13 were the top 10 most abundant genes among the 127 genes identified. The relative abundances (Log_2_ transformed) of the identified CAZyme genes in each sample were shown in Fig. 5A and B.

Comparison of the CAZy families’ profiles using PCoA plot revealed a clear separation between herbivores and omnivores + carnivores, of which the species from the latter two groups were largely overlapping (Fig. 3A). On the other hand, detritivorous crabs partially overlapped with both herbivores and omnivores + carnivores clusters. Results from the hierarchical clustering were largely consistent with the PCoA plot (Fig. 4A). Herbivorous crabs (except one *Parasesarma continentale* sample) formed a cluster with detritivorous *Metaplax* spp., while specimens of the other species in group D (except one *T. arcuata*) clustered with the species from groups O and C (Fig. 4A). For annotations based on KO entries, there was more overlapping between samples from different dietary groups in the PCoA plots, and the distribution was more scattered in the hierarchical clustering tree (Fig. 4B). It is possibly due to the low number of features with only nine KO entries identified, and hence lower resolution among species and samples. PERMANOVA analyses on the identified CAZyme families suggested that the compositions were significantly different among crabs from different dietary groups (FDR adjusted p-value < 0.05) (Supplementary Table S5), even between omnivorous and carnivorous crabs which were highly overlapped in PCoA plot. While for KO entries, only group H was found to be significantly different from the other three dietary groups.

Thirty CAZy families and seven KO were consistently identified as enriched in some metagenomes by at least two DA tools and these genes were mostly found in herbivorous crabs with some of them also observed in detritivores, but none of the CAZyme genes were enriched in omnivorous nor carnivorous crab species (Supplementary Tables S6A and S6B; Fig. 5A and B). In total, 26 CAZyme families and seven KO entries were enriched in at least one of the pairwise comparisons between herbivores and the other three dietary groups. The largest number of enriched features were observed in the herbivores vs. omnivores comparisons (20 CAZyme families, two KO entries), followed by comparison with the carnivorous group (11 CAZyme families, one KO entry) and detritivorous group (three CAZyme families, one KO entry) (Supplementary Table S6). Most of the enriched features related to lignocellulose degradation in herbivorous species were hemicellulose degrading genes (16 CAZyme families, two KO entries) and some pectin degrading (eight CAZyme families, three KO entries) and cellulose degrading genes (two CAZyme families, two KO entries) were also found to be enriched in herbivores. These genes covered the metabolic pathway of debranching, backbone degradation and oligosaccharide degradation of basically all major components of lignocellulose biomass except lignin. For detritivores, six CAZyme families (CE 7, GH 10, 16, 27, 43_9, 43_34) and one KO entry (K01190) related to hemicellulose degradation were enriched over omnivorous crabs and GH43_33 was enriched over carnivorous crabs. These genes cover debranching, backbone degradation, and oligosaccharide degradation of beta-glucans, xylans and mannans. Furthermore, two gene families related to pectin degradation (GH 105 and PL 1_2) were also enriched over group O.

### Taxonomic origin of lignocellulose degrading genes

Proteobacteria contributed to an average of 44% of the lignocellulose degrading genes across all crab samples analyzed (Fig. 6E) and was the dominant phyla in most crab metagenome in all five enzyme categories (degradation of the four lignocellulose compounds and binding) when they were counted individually. A considerable proportion of the microbial CAZyme genes (15%) were from unclassified taxonomic origin (Fig. 6E). Bacteroidetes, Firmicutes, Actinobacteria, Tenericutes and Planctomycetes contributed to an average of 15%, 9%, 6%, 6% and 2% of the lignocellulose degrading genes, respectively. Binding genes and genes related to cellulose, hemicellulose and pectin degradation were of similar taxonomic composition, of which Proteobacteria and Bacteroides accounted for over 50% of genes identified. However, all the lignin-degrading genes were originated from Proteobacteria and Planctomycetes only.

When crabs from different dietary groups were investigated separately, the inter-relationship of bacterial phyla and enzyme categories of herbivores (Fig. 6A) and detritivores (Fig. 6B) were akin to the average across all samples (Fig. 6E) with subtle differences. Herbivores were characterized by a relatively higher proportion of Actinobacteria, contributing to an average of 15% of lignocellulose degrading genes, compared to approximately 5%, 1% and 0% observed in D, O and C, respectively. In detritivores, some lignocellulolytic genes were from Planctomycetes and Spirochetes but not Tenericutes. The profile in omnivores was characterized by the domination of Proteobacteria with an average of 63% compared to 46% in group H and 39% in group D (Fig. 6C). While in carnivores, only 22% of lignocellulose degrading genes were from Proteobacteria but an increase in the proportion of genes from Tenericutes and of unclassified taxonomic origin was observed (Fig. 6D).

### Microbial genes for nitrogen metabolism in mangrove crab gut microbiome

**Fig. 7:**
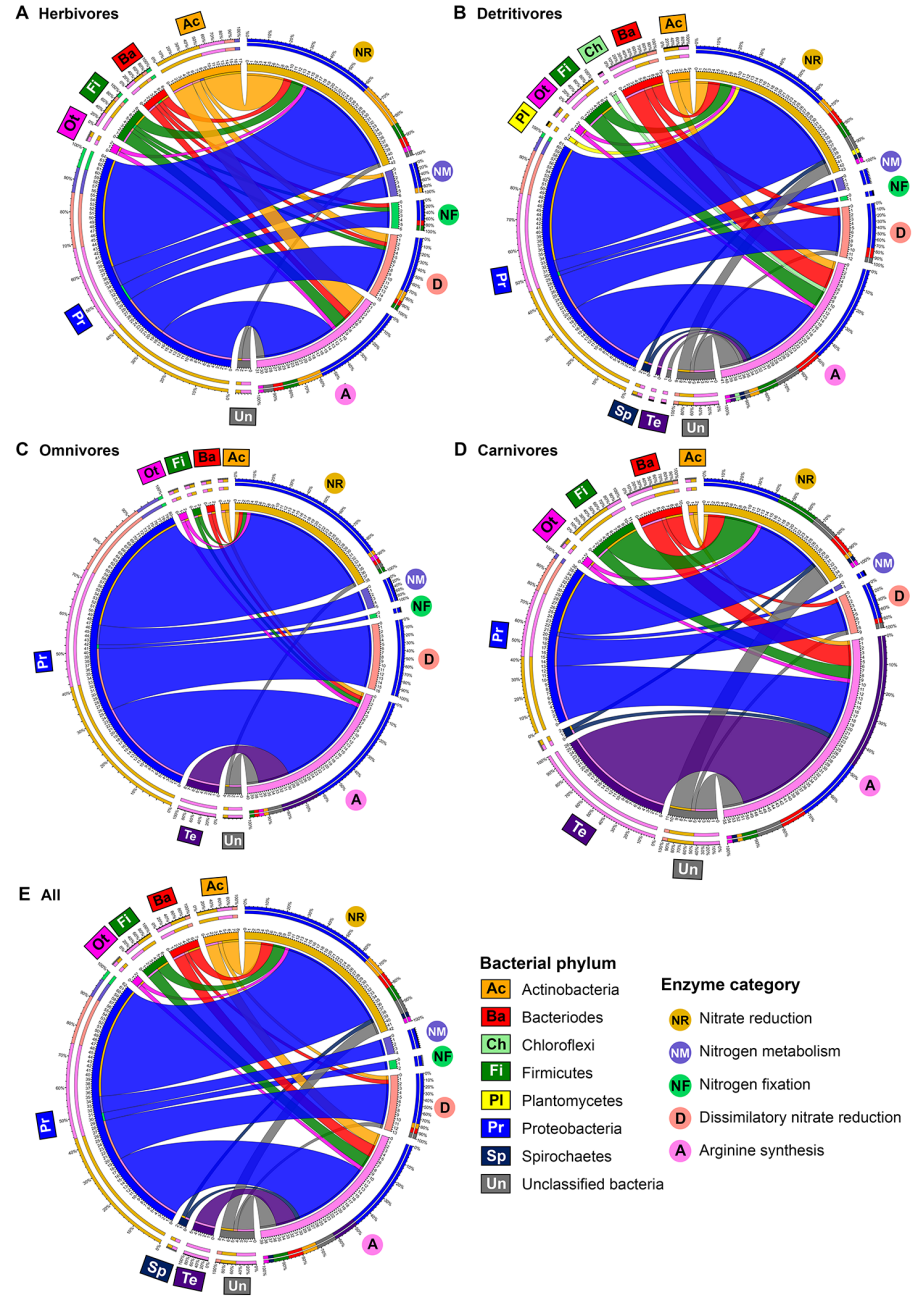
Chord diagrams showing the inter-relationship between bacterial phyla and enzyme categories in nitrogen metabolism-related enzymes identified from KEGG Orthology database in **(A)** herbivores (*n* = 12), **(B)** detritivores (*n* = 18), **(C)** omnivores (*n* = 7), **(D)** carnivores (*n* = 6) and **(E)** all samples (*n* = 43)

The prokaryotic reads from the crab metagenomes recovered 32 out of 42 KO entries related to nitrogen metabolism functions of interest (Supplementary Table S2), including three related to nitrate reduction, four related to nitrogen metabolism, three related to nitrogen fixation, nine related to dissimilatory nitrate reduction, ten related to arginine synthesis, and three related to glutamate synthesis genes. Genes classified under categories of nitrate reduction (42%) and arginine synthesis (39%) together comprised > 80% of the microbial nitrogen metabolism gene while dissimilatory nitrate reduction, nitrogen metabolism and nitrogen fixation composed an average of 13%, 4% and 2% of the annotated nitrogen metabolism gene reads, respectively (Fig. 7E). Glutamate synthesis genes were also found but composed < 1% of the reads annotated to nitrogen metabolism functions and were therefore not shown in the figure.

The abundance of the nitrogen metabolism genes showed significant differences among species and dietary groups (Fig. 5D). PCoA plot (Fig. 3D) and hierarchical clustering tree (Fig. 4D) based on the composition of nitrogen metabolism-related genes demonstrated significant differentiation between groups H and C, while samples from D and O were clustered together and overlapped with both C and H. PERMANOVA analyses revealed significant differences in nitrogen metabolism-related enzyme composition between the four dietary groups (FDR adjusted p-value < 0.05; Supplementary Table S5). Ten KO entries were identified as differentially abundant by at least two DAA tools across all pairwise comparisons between dietary groups, with nine of them depleted in carnivorous crabs (Supplementary Table S6D). Among these nine KO entries, seven nitrogen metabolism-related entries were depleted when compared to herbivores, classified under dissimilatory nitrate reduction (K00362), nitrate reduction (K10534), nitrogen fixation (K02586, K02587, K02588) and nitrogen metabolism (K15576, K15578). Three KO entries classified under dissimilatory nitrate reduction (K00371), nitrogen fixation (K02586) and nitrogen metabolism (K02575) were enriched in detritivores over carnivores, with K02586 being the only KO enriched in both herbivores and detritivores compared to carnivores. K15876 classified under dissimilatory nitrate reduction was the only gene of differential abundance in the comparison between group H and group D (Fig. 5D, Supplementary Table S6D). These genes participate in the conversion of nitrates and atmospheric nitrogen into ammonia. This would potentially increase the nitrogenous nutrient content available for assimilation with the high abundance of genes for arginine and glutamine synthesis from ammonia.

### Taxonomic origin of genes related to nitrogen metabolism

Most of the nitrogen metabolism-related genes originated from Proteobacteria (averaged 62%), followed by Actinobacteria (8%), Bacteroidetes (7%), Firmicutes (6%), Tenericutes (5%), and Spirochetes (2%) (Fig. 7E). 8% of the annotated genes belonged to unclassified prokaryotes. Genes classified under the categories of nitrogen metabolism and nitrogen fixation were contributed predominantly by Proteobacteria in most of the crab species with the remaining phyla altogether only composed of less than 2% in these functions. The genes for nitrate reduction, arginine synthesis and dissimilatory nitrate reduction also mostly originated from the Proteobacteria, but other phyla also contributed a small proportion in these three categories in the crab metagenomes. Tenericutes, on the other hand, only contribute to arginine synthesis.

Variations in the composition of bacterial phyla of nitrogen metabolism-related genes were observed across dietary groups (Fig. 7). The proportion of Actinobacteria was larger in herbivores (17%) than in the other groups (5% in D, 2% in O and 2% in C). In omnivores, Proteobacteria dominated the taxonomic profile of nitrogen metabolism-related genes, contributing up to 80%, while groups H and D showed lower relative abundance with approximately 60% in both groups. Carnivores were characterized by the reduction in proportion of Proteobacteria with only 36% of genes related to nitrogen metabolism. Furthermore, Tenericutes was highly abundant in C (26%) but it was absent in H and low in relative abundance in D (1%) and O (8%).

## Discussion

This study represents the first comprehensive investigation of the enzyme repertoire involved in lignocellulose degradation and nitrogen recycling in mangrove herbivorous crabs. A wide coverage of 43 metagenomes from 23 species with different familial affiliations and dietary preferences allow us to conduct a thorough investigation on mangrove crab microbiome composition and their functional role in lignocellulose degradation based on a comparative approach. The results would also comprehend our understanding of the composition and function of gut microbes from marine crustaceans or even invertebrates which are poorly studied despite their high species diversity.

### Composition and lignocellulose degradation capacity of the mangrove crab gut symbiotic microbiome

Functional metagenomic studies on crustaceans, including crabs, are limited to a few species (e.g. terrestrial isopods, [66]; shrimp [67]) and species with commercial importance (e.g. Chinese mitten crab *Eriocheir sinensis* [28, 29]; mud crab *Scylla* spp. [30, 31]). For the other crustaceans, most of the research effort was focused on taxonomic profiling using 16S amplicon sequencing that provided only little insight into the functional aspect of the microbes. Nevertheless, 16S sequencing data suggests high microbial diversity in crustacean digestive tract and phenotype prediction indicates some of the microbial symbionts may contribute to lignocellulose degradation and/or nitrogen fixation in mangrove herbivorous crabs [32, 33]. Our current attempt represents the first systematic attempt to understand the gut microbiome composition and function in crabs and our results show that Proteobacteria was the most abundant phyla in the mangrove crab metagenome sequences, making up half of the gut microbes in most of the species, followed by Bacteroidetes, Actinobacteria and Firmicutes. The results are largely coherent with previous taxonomic profiles reported in other marine crab species based on shotgun metagenomic sequencing (e.g. *Scylla* [31]) and 16S metabarcoding analyses (deep-sea hydrothermal vent crab *Austinograea* sp. [68] ; freshwater crab *Sinopotamon planum* [69]) of which three mangrove sesarmid species [32] and fiddler crabs [32] were also characterized. Although the alpha diversity measures of richness and evenness were not significantly different across dietary groups or species, the taxonomic profiles of the four dietary groups were distinct from each other. Yet no obvious clustering according to taxonomy (family or genus) nor dietary preference based on the similarity of bacterial phyla composition. Future studies based on a larger sample size will be needed to investigate how phylogenetic and environmental factors (e.g., habitat preference) affect the microbial profile in crab gut microbiome.

We demonstrated a high diversity of microbial CAZyme families in the mangrove crab gut microbiome which is comparable to that reported in other herbivorous arthropods (e.g. termites [58], isopods [22, 66]). All crab species analyzed in this study possess comparable diversity of microbial lignocellulolytic genes, regardless of the dietary composition or taxonomic affiliation, suggesting the microbiome of carnivorous and omnivorous crabs also contain the capacity of degrading lignocellulose to some extent. The most abundant lignocellulolytic genes, including CE 4, 8 xylan esterases, GH 1, 2, 3 glucosidases, GH 12, 26, 51, 55 glucanases, GH 6, 43 xylanases, GH 4, 31, 36 galactosidases, GH 28 polygalacturonidase, and AA 1, 3 lignin oxidases, are found in all studied crabs. These enzymes cover the reactions in the complete degradation pathway of lignocellulose, including debranching, backbone degradation and oligosaccharide degradation enzymes, as well as all enzymes targeting the four major components, cellulose, hemicellulose, lignin, and pectin in plant fiber. This provides strong evidence that gut microbes play a role in the lignocellulose degradation of the mangrove leaf and algae consumed by marine and semi-terrestrial crab species. It is slightly surprising that the repertoire of lignocellulose degrading CAZymes was found in carnivorous crab species as well. This might be attributed to the typically omnivorous lifestyle of crabs with ingestion of algal matter was also reported in the predominantly carnivorous species collected in subtidal areas in this study (*Thranita danae, Scylla paramamosain*, *Epixanthus frontalis* [70–72], which facilitate the acquisition and maintenance of lignocellulolytic microbes in carnivorous species. These results together with previously reported cellulase expression in crab transcriptomes [18, 19] suggest that the crabs implement a mixed mode of digestion utilizing both endogenous and microbial enzymes in lignocellulose degradation, as observed in most of the more advanced herbivorous invertebrate species [11].

Common lignocellulolytic bacterial phyla Proteobacteria, Bacteroidetes, Firmicutes, and Actinobacteria make up the largest part of the lignocellulolytic genes in the crab gut microbiomes. A similar profile is also observed in terrestrial isopods of which the lignocellulose degrading CAZymes are dominated by Proteobacteria, followed by Bacteroidetes, and a small proportion of Actinobacteria and Firmicutes [66]. Compared to the termites which are well-studied for their lignocellulolytic endosymbionts, crabs shared some major lignocellulolytic bacterial phyla with higher termites, including Firmicutes, Proteobacteria, Bacteroidetes and Actinobacteria [24, 58, 73]. The relative importance of the bacterial phyla is, however, differently significant, with Fibrobacteres, Spirochetes, and/or Firmicutes are a major source of prokaryotic cellulose and hemicellulose degradation in higher termites [25, 26, 58, 74] while Fibrobacteres and Spirochetes are also dominant lignocellulolytic microbes in wood-feeding termite [26]. Yet Spirochetes and Fibrobacteres account only for a very minor proportion of microbial lignocellulolytic genes (less than 2% of reads) in crabs and the contribution from Proteobacteria becomes more important (average of 44% across crab species). Hence, the lignocellulolytic symbiont composition of crabs displays higher similarity to the terrestrial isopod that also belongs to Malacostraca crustaceans, than to insects in the Hexapoda.

### Enrichment of microbial cellulolytic capacity in mangrove herbivorous crabs

Comparison of gut microbiome of mangrove crabs in the four dietary groups, herbivores, detritivores, omnivores, and carnivores reveals that although the lignocellulolytic enzymatic repertoire are present in all mangrove crab species analyzed here, but their abundance differs significantly in respect to the dietary preference and composition. Herbivorous and detritivorous species are distinct from the omnivorous and carnivorous species in term of microbial CAZyme profiles, while the profiles are highly similar between herbivorous and detritivorous crabs, or between omnivorous and carnivorous crabs comprised of species from distantly related families. This shows that dietary preference is a stronger driver over phylogeny in determining the microbial CAZyme composition and abundance. Differential abundance analyses found many lignocellulolytic genes were enriched in herbivorous and detritivorous species compared to omnivorous and carnivorous species, but not vice versa. The enriched genes in herbivores comprised high completeness of the degrading pathways of cellulose, hemicellulose, and pectin but not for laccases and lytic polysaccharide monooxygenases (LPMOs, AA1, 3), implying that the capacity for lignin degradation is not significantly increased despite of the presumably high lignin content in mangrove leaf. On the other hand, the enriched enzymes in detritivores are less diverse and smaller in number compared to herbivorous species, indicating a positive correlation between microbial CAZyme abundance and the amount of plant matter in the diet. The herbivorous sesarmid species exhibit a mangrove leaf dominated diet while the diet of detritivorous crabs consists of a mixture of decomposing plant detritus, animal parts as well as microphytobenthos in the sediment that contain a lower lignocellulose content [75]. The detritivores enriched genes mainly related to hemicellulose and pectin degradation. The variations in CAZyme composition are also higher among the detritivorous species that partially reflect their diverse microhabitat preference (e.g., more muddy sediment that contains more organic matter versus sandy substratum that contains more animal meiofauna), and hence variations in organic matter content in diet, which is consistent with previous reports on higher cellulase activities in crab species having a greater plant biomass in diet [20, 76]. This provides further support to our hypothesis that endosymbiotic cellulolytic microbes play an important role in lignocellulose degradation in most crab species but their abundance is strongly correlated with dietary preference, and thus enhancing their capacity for digestion of mangrove leaves and plant detritus.

Despite the high similarities in microbial lignocellulolytic gene composition among crabs with the same dietary preference, their microbial taxonomic composition differed significantly, resulting in a discrepancy between taxonomic and functional profiles across the same set of crab species concerned. This high functional redundancy was also observed in terrestrial isopods *Armadillidium vulgare* and *Porcellio* sp. where laboratory and wild lineages share similar proportions of microbial AAs, CEs, and GHs enzymes, but differ in their taxonomic origin [66]. This may suggest that the symbiotic bacterial communities introduced from the environment were selected against by metabolic interactions within their host holobiont instead of identity [77, 78] and the functional redundancy in the symbiotic microbiome may be more common in the nature that warrants further investigation, especially the crab microbiome will be affected by other environmental factors, including geographic location, salinity as well as microhabitat preference, etc. Our present sampling effort can only explore the effect of diet, so sampling from boarder geographic coverage, habitat and microhabitat would be needed to investigate the influence of other environmental parameters.

### Microbial contribution of nitrogen supply in mangrove herbivorous crabs

The Carbon to Nitrogen (C:N) ratio in mangrove detritus (~ 100) is significantly higher than that of algae (7–10), microphytobenthos (6.4–7.8) and animal tissues (3.7–5.8), so how the mangrove herbivorous crabs acquire sufficient nitrogen from a mangrove leaf-dominated diet represents another major question in mangrove crab ecology [79–81]. Recent stable isotope analysis on crab nutrient sources showed that the contribution of nitrogen from the mangrove litter, and hence the dependence on the additional nitrogen supply is highly variable among mangrove crab species, even amongst different herbivorous species from the family Sesarmidae [82]. For instance, up to half of the nitrogen intake of sesarmid crab *Episesarma versicolor* originated from mangrove leaf litter, but only as low as 8% and 32% of assimilated nitrogen was supplied by the mangrove leaf litter in the sesarmid species *Parasesarma affine* and *P. continentale* (as *P. bidens* in the study). Other mangrove-associated omnivorous and detritivorous crab species obtained less than 25% of their nitrogen from leaf litter, suggesting that most of the mangrove crab species depend heavily on other non-mangrove sources for nitrogen supply. Occasional consumption of animal tissue through predation and/or scavenging [81, 83], intake of more nitrogen-rich supplementary food such as microphytobenthos (MPB) [84, 85] and contribution from the endosymbiotic microbes are invoked as supplementary nitrogen sources for mangrove herbivorous crab species [23, 86], yet empirical evidence to determine the contribution these potential sources remain limited.

Our metagenomic analyses provide the first direct evidence of the presence of microbial genes in dissimilatory nitrate reduction, nitrogen fixation and synthesis of amino acid (e.g., glutamine, arginine), depicting the capability of the gut microbiome to convert ammonia generated by nitrate reduction and fixation to amino acids that can be utilized by both the host and microbes in various mangrove crab species studied, regardless of dietary preference. This finding is consistent with the previous report of nitrogenase activity and enrichment of microbes with nitrogen fixing potential based on 16S metabarcoding analyses on intestinal bacteria of sesarmid species *Episesarma versicolor* and *Neosarmatium smithi* [32]. Recent study also found that the gill-associated bacteria of mangrove fiddler crab can convert ammonia to amino acids [87]. Therefore, the accumulating evidence together suggests symbiotic microbes can contribute at least part of the nitrogen to their host and facilitate their adaptation to semi-terrestrial life.

Furthermore, the herbivorous and detritivorous crabs that exhibit a diet rich in plant matter are enriched in microbial nitrogen metabolism genes compared to carnivorous crabs. Nine out of ten nitrogen metabolism genes that are found to be differentially more abundant between dietary groups belong to the comparison between herbivorous vs. carnivorous and detritivorous vs. carnivorous comparisons. The herbivore-enriched genes include nitrogen transporters (K15576, K15578), nitrate/nitrite reductase (K10534, K00362), nitrogenases (K02586, K02588, K02594) that could facilitate the increase in production of ammonia from atmospheric nitrogen and nitrate reduction. On the other hand, there is no significantly enriched nitrogen metabolism gene detected in the comparisons between omnivorous crabs and crabs from the other three dietary categories, suggesting an intermediate abundance of nitrogen metabolism bacteria in omnivorous crabs. Hence, these results reflect the inverse correlation between the nitrogen content in the diet and the abundance of nitrogen metabolism gut bacteria in mangrove crabs, and the role of endosymbiotic microbes in supplementing nitrogen to the herbivorous crab to fulfill their metabolic need.

## Conclusion

Comparison of bacterial functional abundance found in gut content between crab species under four major dietary preference groups revealed 30 CAZyme families and seven KOs responsible for lignocellulose degradation with significantly higher abundance in herbivorous or detritivorous crabs compared to omnivorous or carnivorous crabs, as well as nine KOs responsible for nitrogen metabolism with significantly higher abundance in herbivorous or detritivorous crab compared to carnivorous crabs. The results demonstrated the potential role of gut microbiome in crab digestion of plant materials and enrichment of nitrogenous nutrients. The dietary groups of crabs are also shown to be more distinct in lignocellulolytic functional profile than the taxonomic profile, showing possibilities of functional selection of the crab gut microbiome by metabolic interactions in the gut. These findings illustrate the potential roles of bacterial symbionts in crab lignocellulose digestion and its significance to both adaptation to an herbivory lifestyle and the mangal terrestrial environment.

### Electronic supplementary material

Below is the link to the electronic supplementary material.

Supplementary Material 1

Supplementary Material 2

## Data Availability

Raw data of all metagenomes are available at NCBI Sequence Read Archive (SRA) with BioProject accession number PRJNA1017629, accessible at https://www.ncbi.nlm.nih.gov/bioproject/PRJNA1017629. The assembly of the gut metagenome of each species as well as the unnormalized read counts tables on bacterial phyla and functional features in CAZy and KEGG databases are available at DRYAD, accessible at https://datadryad.org/stash/share/jGokZ8PmSAfDhjV_iAN4UgVWlgFqh4pqDGrEcY8GW30.

## Abbreviations

AA: Auxiliary Activities
CAZy/CAZyme: Carbohydrates Active enZyme
CBM: Carbohydrates Binding Modules
CE: Carbohydrate Esterase
DA/DAA: Differential Abundance/ Differential Abundance Analysis
FDR: False Discovery Rate
KEGG: Kyoto Encyclopedia of Genes and Genomes
KO: KEGG Orthology
LPMO: Lytic Polysaccharide Monooxygenases
RefSeq nr database: NCBI Reference Sequence non-redundant protein database
ORF: Open Reading Frame
PCoA: Principal Coordinate Analysis
PERMANOVA: Permutational Multivariate ANOVA
PL: Polysaccharide Lyase
SRA: The Sequence Read Archive
