# Supplementary material for: Metagenomic analysis of gut microbiome illuminates the mechanisms and evolution of lignocellulose degradation in mangrove herbivorous crabs
Source: BMC Microbiol. 2024 Feb 13;24:57. doi: 10.1186/s12866-024-03209-4 (PMC10863281; doi:10.1186/s12866-024-03209-4)
Supplement: Supplementary file 2 — Supplementary Material 2 [file 12866_2024_3209_MOESM2_ESM.pdf]

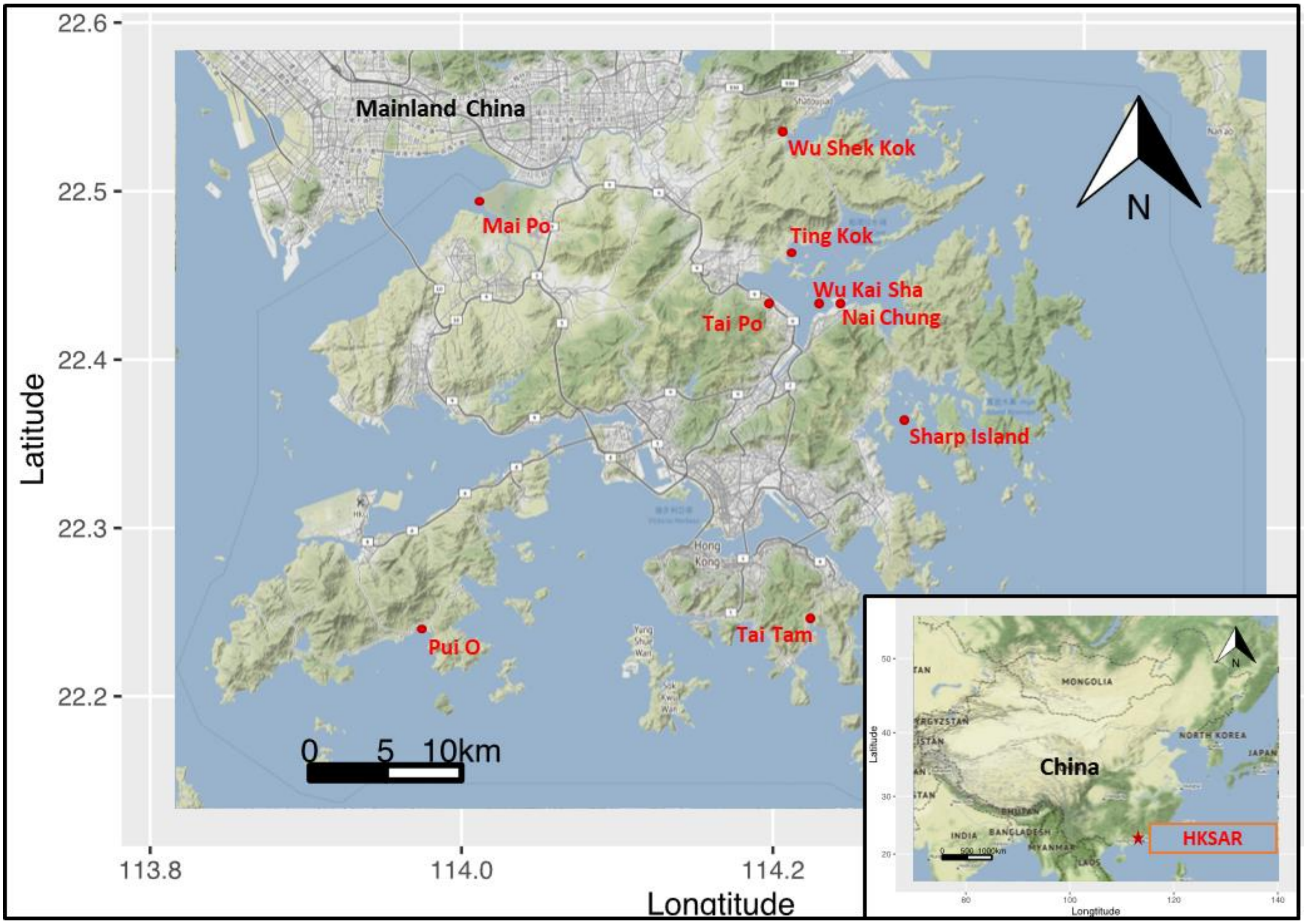

Supplementary Figure 1. Locations of sample collection sites. Adapted from map tiles created by Stamen Design, under CC BY 4.0. Data by OpenStreetMap, under Open Database License.

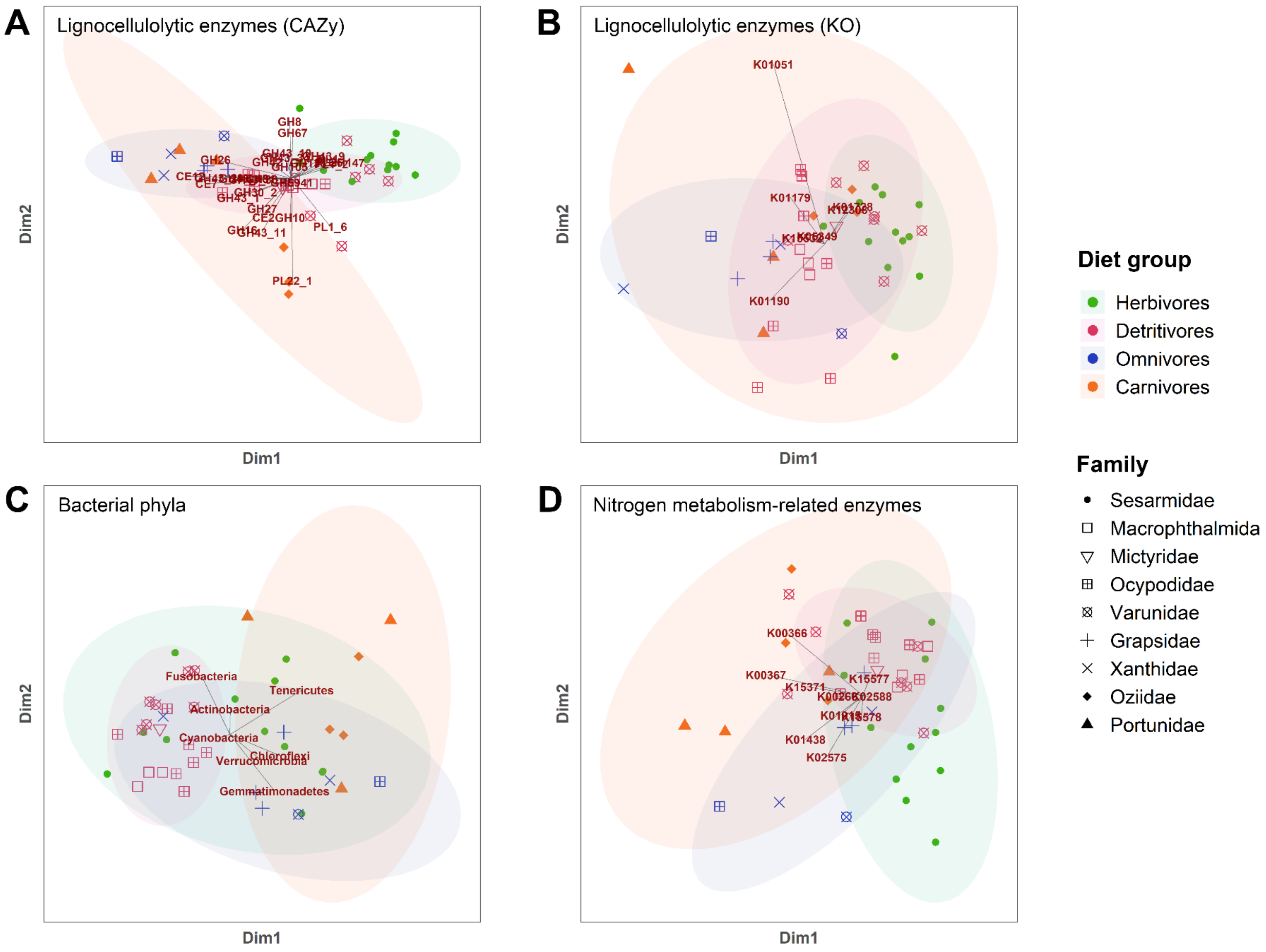

Supplementary Figure 2. PCA plots of differentially abundant features. (A) Lignocellulolytic enzymes annotated to CAZy families, (B) lignocellulolytic enzymes annotated to KEGG Orthology entries, (C) bacterial phyla and (D) nitrogen metabolism related enzymes annotated to KEGG Orthology entries. Each point corresponds to a sample and the symbol represents the family to which the sample belongs. Labels in red are features with significant differences in at least one pairwise comparison among the dietary groups identified with consensus from ALDEx2, ANCOM-BC and DESeq2.
